# Cardiac thrombus dissolution in acute ischemic stroke: A substudy of Mind the Heart

**DOI:** 10.1016/j.heliyon.2023.e20627

**Published:** 2023-10-04

**Authors:** C.F.P. Beemsterboer, L.A. Rinkel, V. Guglielmi, N.-S. Groeneveld, N.H.J. Lobé, S.M. Boekholdt, B.J. Bouma, F.F. Muller, L.F.M. Beenen, H.A. Marquering, C.B.L.M. Majoie, Y.B.W.E.M. Roos, A. van Randen, R.N. Planken, J.M. Coutinho

**Affiliations:** a Department of Neurology, Amsterdam UMC, Location AMC, the Netherlands; b Department of Radiology and Nuclear Medicine, Amsterdam UMC, Location AMC, the Netherlands; c Department of Cardiology, Amsterdam UMC, Location AMC, the Netherlands; d Department of Biomedical Engineering and Physics, Amsterdam UMC, Location AMC, the Netherlands

## Abstract

**Background:**

Cardiac thrombi are an important cause of ischemic stroke but are infrequently detected on cardiac imaging. We hypothesized that this might be explained by early dissolution of these cardiac thrombi after stroke occurrence.

**Methods:**

We performed a single-center observational pilot study between November 2019 and November 2020, embedded in the larger “Mind-the-Heart” study. We included patients with AIS and a cardiac thrombus in the left atrium or ventricle (filling defect <100 Hounsfield Units) diagnosed on cardiac CT that was acquired during the initial stroke imaging protocol. We repeated cardiac CT within one week to determine if the thrombus had dissolved.

**Results:**

Five patients (four men, median age 52 years, three with atrial fibrillation and one with anticoagulation therapy at baseline) were included. Median time from symptom onset to first cardiac CT was 383 (range 42–852) minutes and median time from first to second cardiac CT was three days (range 1–7). Two patients received intravenous thrombolysis (IVT). In total, six thrombi were seen on initial CT imaging (one in the left ventricle, four in the left atrial appendage, one in the left atrium). The left atrium thrombus and one left atrial appendage thrombus had dissolved on follow-up cardiac CT, one of which was in a patient with IVT treatment.

**Conclusion:**

This pilot study illustrates that cardiac thrombi can dissolve within days of stroke occurrence both with and without IVT treatment.

## 1 Introduction

Cardiac thrombi are an important source of embolism causing ischemic stroke [1]. Detection of a cardiac thrombus can affect patient management in patients with no other indication for anticoagulation therapy. Moreover, patients with a cardiac thrombus may have a higher risk of recurrent stroke and might benefit from early initiation of anticoagulant treatment [2]. Guidelines recommend echocardiography as a first-line screening method for cardiac thrombi in stroke patients [1]. While transoesophageal echocardiography (TEE) has a higher diagnostic yield compared to TTE [3], and is the gold standard to detect thrombi situated in the left atrium [4], TEE is invasive and may require sedation and can therefore challenging to acquire, especially in the acute phase of ischemic stroke. Therefore, transthoracic echocardiography (TTE) is most commonly used for screening [5], but the yield of TTE to detect cardiac thrombi, especially those in the left atrial appendage, is low [6]. Recent studies have shown that cardiac CT, acquired during the acute stroke imaging protocol, is technically feasible, has a higher diagnostic yield for cardiac thrombi and is a suitable alternative to TTE to screen for cardiac sources of embolism in patients with ischemic stroke [7,8]. The higher yield for cardiac thrombi on cardiac CT is likely due to its superior capability to visualize the left atrial appendage, but may also be due to dissolving cardiac thrombi which are missed on TTE as this is often performed days or weeks after the stroke for logistical reasons. There are no data on the dissolution rate of cardiac thrombi in the first days after ischemic stroke. This information would both increase our understanding of the pathophysiology of cardioembolic stroke and would help inform the optimal timing of cardiac imaging after stroke to detect cardiac thrombi. We therefore aimed to assess if cardiac thrombi diagnosed in the acute phase of stroke on cardiac CT dissolve during the first days after ischemic stroke.

### 1.1 Methods

This pilot study was a substudy of Mind the Heart, which is a prospective single-center cohort study performed in Amsterdam UMC, a comprehensive stroke center in the Netherlands. Consecutive adult patients with acute ischemic stroke underwent prospective ECG-gated cardiac CT during the initial stroke imaging protocol, immediately following non-contrast-enhanced CT of the brain, CT perfusion, and non-gated CT-angiography of the aortic arch, cervical and intracranial arteries. Patients also underwent routine stroke work-up, including TTE. Patients were included from May 2018 to November 2020 [8,9].

For the current substudy, we included AIS patients with a cardiac thrombus detected on initial cardiac CT. Included patients underwent repeat cardiac CT according to the same scanning protocol within one week. Cardiac CT images were assessed by a cardioradiologist. Patients underwent TTE as soon as possible, which was evaluated by a cardiologist. During assessment, the cardiac radiologist and cardiologist were blinded to each other's scoring.

Radiological findings were scored using predefined criteria [9]. On cardiac CT, a thrombus was defined as a low-attenuated mass <100 Hounsfield Units in the left ventricle, left atrium or left atrium appendage [9]. On TTE, a thrombus was defined as a circumscribed echogenic or echolucent mass in the left ventricle, left atrium or left atrium appendage, distinct from the surrounding wall [9].

We assessed the frequency of dissolution of cardiac thrombi on sequential cardiac CT compared to initial CT.

Mind the Heart was approved by the medical ethics committee of Amsterdam UMC, location AMC (METC:2018 017,NL64139.018.18). The amendment for this substudy was approved in November 2019. All patients or representatives provided written consent.

## 2 Results

Between November 2019 and November 2020, a cardiac thrombus was detected on initial CT in 14 patients. Follow-up cardiac CT could not be obtained in nine patients (no informed consent in one, discharge within 24 hours in six, logistical issues in one, and deterioration of clinical condition in one patient). Therefore, follow-up cardiac CT was performed in 5 patients (4 men, median age 52 years [range 50–84]). Two patients had a history of atrial fibrillation (AF) and one patient was newly diagnosed with AF during hospital admission. One patient used anticoagulation therapy at baseline. Two patients were treated with IVT and both received IVT after the initial cardiac CT. The reasons for not administering IVT were anticoagulation use in one patient and presentation outside of the recommended time-window in two patients. The median time from symptom onset to first cardiac CT was 383 (range 42–852) minutes and median time between first and second cardiac CT was three (range 1–7) days (Table 1). Additional baseline characteristics of included patients are presented in Table 2.

**Table 1:** Clinical and imaging data.

Patient	Age	Sex	ACT at baseline^a^	AF	IVT	Days between first and second CT	First cardiac CT findings	Second cardiac CT findings	Days between first CT and TTE	TTE findings	Days between first CT and ACT	Type and dose ACT
**1**	50	M	–	–	+	1	LV thrombus	LV thrombus	1	LV: thrombus	1	Therapeutic Fraxiparine 7600 IU twice a day
**2**	55	M	–	KAF	+	6	LA thrombus LAA thrombus	LA thrombus dissolved LAA thrombus	6	No thrombus	2	Dabigatran 150 mg twice a day
**3**	84	V	+	KAF	–	1	LAA thrombus	LAA thrombus	0	No thrombus	7	Warfarin once a day (dose based on INR)
**4**	52	M	–	–	–	3	LAA thrombus	LAA thrombus	0	No thrombus	8	Warfarin once a day (dose based on INR)
**5**	70	M	–	AFDAS	–	7	LAA thrombus	LAA thrombus dissolved	2	No thrombus	5	Dabigatran 150 mg twice a day

ACT: anticoagulation therapy, IVT: intravenous thrombolysis, TTE: transthoracic echocardiography, LV: left ventricle, LA: left atrium, LAA: left atrial appendage, AF indicates atrial fibrillation, KAF indicates known AF, AFDAS indicates AF diagnosed after stroke.

*switched to Warfarin after two weeks.

The patient with a LV thrombus has no known history cardiac disease upon presentation, but further work-up revealed akinesia of the apex, believed to be possibly resulting from an old myocardial infarction although Takotsubo myopathy could not be excluded.

**Table 2:** Additional baseline characteristics of included patients.

	Patient				
	1	2	3	4	5
Systolic blood pressure – mmHg^a^	162	172	200	119	128
NIHSS score	6	18	18	1	3
Medical History					
Previous ischaemic stroke	–	–	–	–	–
Transient ischaemic attack	–	–	–	–	–
Atrial fibrillation	–	+	+	–	–
Diabetes mellitus	–	–	+	–	–
Hypertension	–	–	+	–	–
Hypercholesterolemia	–	–	–	–	–
Smoking	–	–	–	–	–
Myocardial infarction	–	–	–	–	–
Other cardiac history	–	Myocarditis	–	Endocarditis	–
Medication use at baseline					
Anticoagulation	–	–	+	–	–
Antiplatelet	–	–	–	–	–
Anti-hypertensive drugs	–	–	+	–	–
Statin	–	–	–	–	–
Lab results at baseline					
APPT - seconds	23	25	NA	21	27
INR	1.06	NA	2.6	NA	1.11
GFR - mL/min/1.73 m^2^	83	60	61	49	86
Thrombocyte count - × 10^3^ per mm	382	212	216	686	210
Glucose - mmol/l	5.1	5.8	14.6	11.1	7.7
Intracranial large vessel occlusion	–	+	+	–	–
Endovascular thrombectomy	–	+	+	–	–
Baseline CHA₂DS₂-VASc Score	0	0	5	0	1
Baseline HASBLED score	1	1	2	1	1

NIHSS indicates National Institute of Health Stroke Severity, APPT indicates Activated Partial Thromboplastin Time, INR indicates international normalised ratio, GFR indicates glomerular filtration rate.

**Fig. 1:**
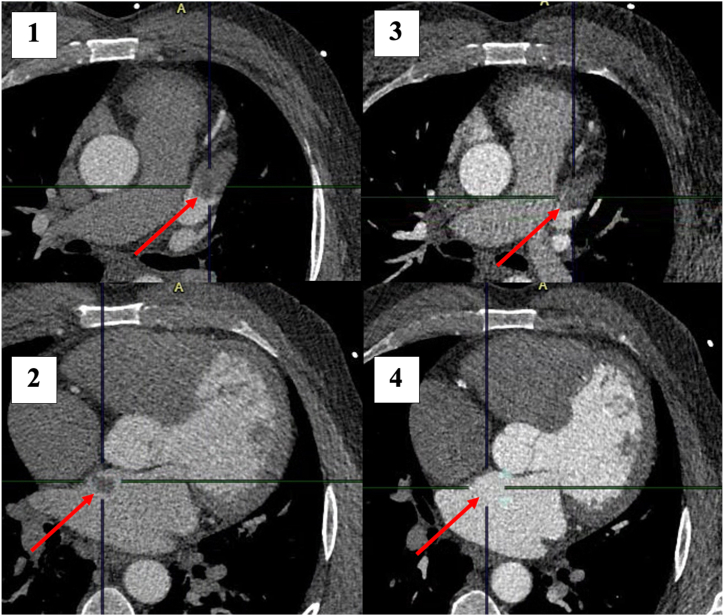
Patient with a left atrial appendage (1) and left atrial thrombus (2) on cardiac CT acquired during the initial stroke imaging protocol. Cardiac CT repeated after six days, showed a persisting left atrial appendage thrombus (3) while the left atrial thrombus was dissolved (4).

Six thrombi were seen on initial cardiac CT (left ventricle [LV] 1, left atrium [LA] 1, left atrial appendage [LAA] 4, Table 1). One patient had two thrombi (Fig. 1 [1–4]). On follow-up CT 2/6 (33%) thrombi had dissolved: one in the LA and one in the LAA (Table 1). In the patient with two thrombi, the LA thrombus was no longer visible on follow-up imaging while the LAA thrombus persisted. Of the two patients treated with IVT, a cardiac thrombus had dissolved in one. Four patients were on anticoagulant therapy at the time of follow-up cardiac CT, including both patients in whom the cardiac thrombus had dissolved.

TTE was performed between initial and follow-up cardiac CT with a median time between first CT and TTE of one day (range 0–6). TTE only showed the LV thrombus.

## 3 Discussion

In this pilot study, two of the six thrombi seen on initial cardiac CT in five patients with AIS had dissolved on follow-up cardiac CT performed within one week.

A case report of a non-stroke patient with AF reported that a LA thrombus dissolved shortly after IVT treatment [10]. We observed that a LA thrombus dissolved following IVT treatment, while a LAA and LV thrombus persisted. However, we also observed resolution of a LAA thrombus within days of stroke occurrence in a patient without IVT. In the patient treated with anticoagulation at baseline, the LAA thrombus did not dissolve. This suggests that thrombi that develop under anticoagulation therapy may be less likely to dissolve in the first days after stroke.

A study in 16 patients with pulmonary embolism and concurrent right atrium thrombi, reported a dissolution rate of 100% on TTE within 24 hours following IVT [11]. This higher dissolution rate may be explained by a difference in pathogenesis in thrombi in the left and right side of the heart [12]. Alternatively, it could be that smaller thrombi were missed on TTE.

A study reporting on two cohorts using echocardiography to determine the dissolution rate of LAA thrombi in non-stroke patients who were treated with anticoagulation found a dissolvement rate of 40–60% within 3–12 weeks [13]. Another study observed a dissolution rate of 79% within one year in patients treated with adequate anticoagulation therapy [14], indicating that LAA thrombi can persist over a longer time in patients despite appropriate anticoagulation.

The mechanism of thrombi which dissolve in the first days after stroke may potentially explain a proportion of the embolic stroke of undetermined source (ESUS) population [15], in which no source of embolism is detected despite adequate work-up while the stroke is suspected to have an embolic origin. Future research in larger cohorts is needed reliably determine how frequently thrombi dissolve in patients with no other clear cause of their stroke to assess to what extend this mechanism plays a role in explaining ESUS cases.

Missing cardiac thrombi may have important clinical implications since up to one third of the patients with a cardiac thrombus on cardiac CT acquired during the acute stroke imaging do not have atrial fibrillation [[8], [16]]. If these thrombi are missed after dissolvement when cardiac imaging is performed too late, this could potentially lead to inadequate secondary prevention in these patients.

Patients with cardiac thrombi -especially persisting thrombi-may have a higher risk of recurrent stroke. Ongoing trials aim to assess whether patients with cardioembolic stroke benefit from early initiation of anticoagulant treatment [2], and it may be that patients with a persisting cardiac thrombus benefit most from early initiation of anticoagulant therapy.

One of the potential challenges of cardiac CT in detecting cardiac thrombi of is to differentiate between a true thrombus in the LAA and filling artefacts. Delayed scanning is known to strongly improve this differentiation [17]. For consistency reasons, the same protocol was used for both cardiac CT scans. However, while the initial cardiac CT was effectively a delayed scan since it was acquired following CT-angiography and CT-perfusion, the follow-up cardiac CT was not. Leaving out delayed scanning during follow-up imaging may have underestimated the number of dissolved thrombi, as this helps differentiating between LAA thrombi and filling artefacts.

The two patients in whom a cardiac thrombus had dissolved on follow-up CT were also those with the longest time difference between the two cardiac CTs. Future studies are needed to determine if there is a distinguishable time trend in dissolution of cardiac thrombi in the first days after stroke.

Strengths of this study are the prospective design and the short time windows between the first and sequential cardiac CT and first CT and TTE. The limited number of patients that are included in this study is a clear limitation. While the true rate at which cardiac trombi dissolve cannot be determined from this study due to the limited sample size, it is the first study to demonstrate that cardiac thrombi can dissolve within the first week after stroke both with and without IVT treatment. Furthermore, we were unable to assess the dissolution of cardiac thrombi beyond the first week, as we did not perform further imaging in patients with a persisting thrombus. Finally, we did not perform TEE in patients, which is known to be superior to TTE and is considered the gold standard for detection of left atrial thrombi [4]. The findings of this study should be confirmed in a larger cohort of patients who also undergo TEE.

## 4 Conclusion

We illustrate that cardiac thrombi can dissolve within days of stroke occurrence both with and without IVT treatment.

## Data availability statement

Data associated with this study has not been deposited into a publicly available repository, but data will be made available on reasonable request.

## CRediT authorship contribution statement

**C.F.P. Beemsterboer:** Data curation, Formal analysis, Investigation, Methodology, Writing – original draft, Writing – review & editing. **L.A. Rinkel:** Conceptualization, Data curation, Formal analysis, Investigation, Methodology, Writing – original draft, Writing – review & editing. **V. Guglielmi:** Conceptualization, Data curation, Methodology, Writing – review & editing. **N.-S. Groeneveld:** Data curation, Writing – review & editing. **N.H.J. Lobé:** Data curation, Writing – review & editing. **S.M. Boekholdt:** Data curation, Writing – review & editing. **B.J. Bouma:** Data curation, Writing – review & editing. **F.F. Muller:** Data curation, Writing – review & editing. **L.F.M. Beenen:** Data curation, Writing – review & editing. **H.A. Marquering:** Data curation, Writing – review & editing. **C.B.L.M. Majoie:** Data curation, Writing – review & editing. **Y.B.W.E.M. Roos:** Data curation, Writing – review & editing. **A. van Randen:** Data curation, Investigation, Writing – review & editing. **R.N. Planken:** Conceptualization, Data curation, Investigation, Writing – review & editing, Formal analysis, Methodology. **J.M. Coutinho:** Conceptualization, Data curation, Formal analysis, Supervision, Writing – review & editing.

## Declaration of competing interest

The authors declare the following financial interests/personal relationships which may be considered as potential competing interests: CBLMM has received research grants from CVON/Dutch Heart Foundation, European Commission, TWIN Foundation, Healthcare Evaluation Netherlands and Stryker (paid to institution). CBLMM, HAM and YBWEMR are shareholders of Nicolab, a company that focuses on the use of artificial intelligence for medical image analysis. JMC reports grants from Medtronic, Boehringer Ingelheim, and Bayer outside the submitted work (paid to institution). JMC and HAM are shareholder of TrianecT. The other authors have no financial conflicts of interest.
